# Expression of cyclooxygenase-2 (COX-2) in epithelial ovarian cancers in an Indigenous African population of Kano, Nigeria

**DOI:** 10.3332/ecancer.2025.1838

**Published:** 2025-02-05

**Authors:** Jimoh Ajanaku Abdulrazaq, Mohammed Abdullahi, Nzekwe Patric Chim, Richard Kelechi Samuel

**Affiliations:** 1 Department of Pathology, Federal University of Health Sciences Azare, Azare 751101, Bauchi State, Nigeria; 2 Department of Pathology, Ahmadu Bello University, Zaria 810211, Kaduna State, Nigeria; 3 Department of Anatomic Pathology, Asaba Specialist Hospital, Okpanam 320108, Delta State, Nigeria; 4 University of Abuja Teaching Hospital, Gwagwalada 902101, Abuja, Nigeria; a https://orcid.org/0000-0003-4052-3300

**Keywords:** COX-2 expression, ovarian cancer, Indigenous Africa population, Kano, Nigeria

## Abstract

The high case-fatality of epithelial ovarian cancer (EOC) stems from the absence of recognisable premalignant lesion, lack of effective screening, advanced stage at presentation, high recurrence and COX-2 over-expression. Expression of COX-2 in EOCs is associated with unfavorable survival outcomes. In Nigeria, younger age affectation, rising incidence and poor survival outcomes of EOC provide the driving forces for researchers in terms of screening, prevention and targeted therapy.

**Methods:**

All the 52 EOC cases over a 5-year period were included, but only 48 formalin-fixed paraffin-embedded tissue blocks were sectioned and stained with COX-2 antibody. COX-2 expression was scored for distribution (no cells = 0, 1%–10% = 1, 11%–50% = 2, 51%–80% = 3, 81%–100% = 4) and intensity (no stain = 0; weak = 1; moderate = 2, strong = 3). The immunoreactive score (IRS) is a product of intensity (I) and distribution (D) as: 9–12 strongly +, 5–8 moderately +, 1–4 weakly + and 0 negative. Over-expression of COX-2 is an IRS of 5–12. Outcomes were statistically evaluated with clinicopathological data.

**Results:**

EOC cases have a mean age of 50.0 years, and peaked in the 6th decade. High-grade serous carcinoma (HGSC) accounted for the majority (50%), followed by low-grade serous carcinoma and mucinous carcinomas each at 17.3%. High-grade carcinomas accounted for 61.5% of cases. Over-expression of COX-2 was observed in 52.1% of the cases with significant associations between COX-2 expression and high-grade EOC, type II EOC or HGSC but not with the other histological sub-type or age.

**Conclusion:**

More than one-third of EOCs occurred ≤50 years and more than half of EOCs over-expressed COX-2. There were significant statistical associations between COX-2 over-expression and grade, type II tumours or HGSC indicating that it may influence the outcomes of EOC with possible variation in tumour type and grade.

## Introduction

Globally, epithelial ovarian cancers (EOCs) are lethal diseases and have the highest case-fatality ratio of all gynaecological malignancies [1,2]. The EOCs high mortality stems from the absence of recognisable premalignant lesions, lack of effective screening and early detection strategies, advanced stage at presentation, COX-2 overexpression and the rapid emergence of drug resistance, and these provide the driving forces for researchers [2–4]. The type I EOCs (low-grade serous, mucinous, endometrioid, clear cell carcinomas and malignant Brenner tumour) are believed to arise from extra-ovarian benign lesions while type II EOCs (high-grade serous carcinomas (HGSC), undifferentiated carcinomas and carcinosarcomas) originate from fallopian tube fimbriae carcinomas [5]. Younger age, poor survival rate, inadequate access to healthcare facilities, late presentation, alternative care-seeking behaviour and poor drug compliance characterised the challenges associated with ovarian cancer in Nigeria [3].

Several observations have shown strong associations between EOCs and COX-2 (including its inhibitors such as NSAIDs), and these are evident from its pathological roles as well as genetic, experimental, epidemiological and clinical studies. Over-expression COX-2 is strongly involved in inflammation and carcinogenesis and has unfavourable outcomes in EOCs [6, 7]. Also, polymorphisms of COX-2 Genes such as 765 CG, 1195 AA genotype and 765 C alleles increase the EOC risk while the AG genotype and G allele of −1,195 gene decrease the risk [6]. Experimental evidences show that COX-2 over-expression is a sufficient single molecule in tumour onset and progression in transgenic mouse models [8, 9]. Evidence has demonstrated that the use of NSAIDs is associated with reduced incidence and mortality of EOC [10]. Compared to women who do not use aspirin, pre-diagnosis and post-diagnosis use of aspirin (NSAIDs) is associated with a reduction in the relative risk of development of EOCs and dying from EOCs, respectively [11–13].

Though the causes of EOCs are unknown, we believe that the association between COX-2 expression and EOCs is strong in terms of EOC pathogenesis, incidence and mortality and this offers an opportunity for targeted therapy and chemo-prevention. However, studies are still needed in this regard. The pattern of COX-2 expression is an important window into the chemo-preventive and targeted therapy of selective COX-2 inhibitors. The use of NSAIDs has been approved for the prevention of certain cancers in the USA and Australia based on the available research data [14, 15]. Unfortunately, such data is sparse in Africa. The findings from this study, therefore, are expected to contribute to the development of molecular targeted therapy and chemo-prevention (with a specific focus on anti-COX-2) for EOC. The aim of this retrospective study is to assess the proportion of EOCs in Aminu Kano Teaching Hospital (AKTH), Kano that over-expressed COX-2 and determines any relationship between COX-2 over-expression with clinico-pathological features such as age, histological subtype and tumour grade.

## Materials and methods

This retrospective hospital-based descriptive study was carried out in the histopathology department of AKTH, Kano, Nigeria, covering cases from 1st January 2015 to 31st December 2019. The hospital is a tertiary hospital with over 700-bed capacity. The histopathology department of AKTH receives an average of 5,500 histological samples per annum and renders services including cytology, histology, autopsy and immunohistochemistry.

Fifty-two cases of EOC sub-types (diagnosed histologically) within the study period in the department whose specimens were primarily from ovaries were included. Only 48 cases of EOCs were suitable for COX-2 immunohistochemistry. Cases with insufficient clinical information, particularly biodata, missing or damaged blocks and tissue blocks with insufficient tissue were excluded. Data, such as age, histologic diagnosis and grade of the cancer was obtained from pathology request forms, patients’ case notes, and duplicate copies of histopathological reports and slide reviews of cases. Patients’ identity was concealed at all times. Ethical approval for this study was obtained from the Health and Research Ethics Committee of AKTH (ethical review reference number: AKTH/MAC/SUB/12A/P-3/V1/2915).

### Immunohistochemistry for COX-2 expression

Immunohistochemical staining was performed using an anti-COX-2 rabbit polyclonal antibody (Elabscience, USA, catalog No. E-AB-70031), used at a 1:500 dilution according to standard immunohistochemical staining protocols. A kidney sample with intact renal tubules was used as a positive control while a negative control was obtained by replacing primary antibody with distilled water.

The slides were viewed under the light microscope and brown cytoplasmic and membranous staining was interpreted as positive staining for COX-2. COX-2 expression was scored semi-quantitatively using the immunoreactive score (IRS), a final score that is a product of the intensity and distribution of the COX-2 immunoreactivity score [16]. The intensity of staining was scored as 0 for no staining, 1 for weak staining, 2 for moderate and 3 for strong staining. The percentage of positive tumour cells was scored: 0 indicating no cell with positive reaction, 1 indicating 1%–10% of cells with positive reaction, 2 indicating 11%–50% of cells with positive reaction, 3 indicating 51%–80% of cells with positive reaction and 4 indicating greater than 80% of cells with positive reaction (Table 1).

The final IRS score obtained by multiplying the distribution and intensity for each tumour was graded as follows: 9–12 strongly positive, 5–8 moderately positive, 1–4 weakly positive and 0 negative. COX-2 was considered over-expressed if the IRS score was moderate to strong (that is a score of 5 to 12).

### Statistical analysis

Statistical analysis was performed using the chi-square test (SPSS Inc, Chicago, IL, USA, version 22) with statistical significance set at *p* < 0.05.

## Results

Fifty-two EOCs were analysed, but only 48 cases had COX-2 immunohistochemistry. The age category of primary EOC patients ranged from 20 to 75 years with a mean age of 50.0 (S. D ± 14.49) years. The largest proportion of cases (35 of 52 cases, 67.3%) clustered within the 40–69 age range which also corresponded to the highest frequency of occurrence of high-grade carcinomas, and peaked in the 6th decade. Twenty-two cases (42.3%) were <50 years while 11.5% (6) cases were ≥70 years (Tables 2 and 3).

The predominant histological type in all age groups was (HGSC, 50%), this was followed by low-grade serous carcinoma (LGSC, 17.3%) and mucinous carcinomas (17.3%). Type I ovarian carcinomas observed in this study accounted for 46.2% (24 cases) and this includes 9 LGSC, 9 mucinous, 3 clear cell, 1 malignant Brenner, 1 carcinosarcoma and 1 transitional cell carcinomas. Type II carcinomas are HGSC (26) and endometrioid carcinomas (2).

Thirty-two (61.5%) EOCs were high grade, 81.3% of these were HGSC, and the remaining 18.8% were clear cell carcinoma (3), endometrioid carcinoma (2) and carcinosarcoma (1) (Tables 2 and 3). The remaining 38.5% of EOCs were LGSC (9) mucinous carcinoma (9), malignant Brenner tumour (1) and transitional cell carcinoma (1).

### COX-2 expression

Over-expression of COX-2 was observed in 52.1% (25 out of 48) of cases: 48% (12 of 25 positive cases) of this was moderately positive, while the remaining 52% was strongly positive (Table 1). For the COX-2 negative cases, the distribution of IRS scores was 34.8% (IRS of 0) and 65.2 (IRS of 1–4). Sixty-eight percent of positive cases (17 of 25) occurred in the 40–69 age group, only (16.7%) one of the six tumours in the 20–29 age category over-expressed COX-2, while 50% of the six cases in the age group 70–79 were positive (Table 4). For histological sub-type, COX-2 over-expression in HGSC, LGSC, mucinous carcinoma, clear cell carcinoma and carcinosarcoma were 69.2% (18 of 26), 22.2% (2 of 9), 33.3% (3 of 9), 50% (1 of 2) and 100% (1), respectively. The respective proportion of types I and II carcinomas (HGSC) that were positive for COX-2 is 31.8% (7 of 22) and 69.2% (18 of 26).

Twenty (69%) of 29 cases of high-grade EOC over-expressed COX-2 which includes 18 cases of HGSC and a case of clear cell carcinoma and carcinosarcoma. By contrast, only 5 out of 19 cases of LGSC over-expressed COX-2 (Table 4). There were significant associations between COX-2 expression and high-grade EOCs (*χ*2 = 8.36676, df 1, *p* 0.0038) or type II sub-type carcinomas (HGSC) (*χ*2 = 6.68351, df 1, *p* 0.0097) but not with the other histological sub-type (*χ*2 = 9.55719, df 5, *p* 0.0888) or age (*χ*2 = 1.77445, df 5, p 0.87939).

## Discussion

### Summary of main results

EOCs affect a large proportion of premenopausal women with a mean age of 50 years, 42.3% of cases occurred at <50 years and 88.5% cases manifested ≤70 years. Serous or type II ovarian carcinomas, and high-grade lesions were the predominant tumours. Overall, 52.1% of EOCs over-expressed COX-2 with significant associations with high-grade EOCs or type II sub-type carcinomas (HGSC) but not with the other histological sub-type, or age.

### Results in the context of published literature

EOC is a lethal disease and demonstrates wide variations in terms of age, histological types and outcomes across regions and races. This index study, it affects relatively younger women. This younger patient affectation has also been documented in other studies in Nigeria, across Africa, India, Asia and Iran [17–22]. By contrast, EOC in the White is a disease of older menopausal women (mean age of 65 in the USA) and peaks in the late 70s compared to the Blacks, Asia and Hispanics [18, 23–27]. This disparity in the pattern of occurrence across races may be explained in part by genetic make-up, risk exposure, lifestyle modification and duration of life expectancy among other reasons. By histological sub-type and grade, high-grade EOCs and serous carcinomas predominate have also been observed across the globe [28].

Over-expression of COX-2 was observed in 52.1% of the EOCs which is in agreement with other studies where the rate of over-expression ranged from 40% to 91% [7, 28, 29]. The wide variation (40% to 91%) in COX-2 over-expression in ovarian cancers in the published studies may be due to study design (all types of ovarian cancer versus EOCs or specific histological sub-type), disease stage, tumour grade and histological sub-type, genetic make-up, patients' characteristics such as age, among other reasons.

For example, Tiina-Liisa *et al* [30] analysed only serous carcinoma and found that 70% of them expressed COX-2 in association with high grade, age greater than 57 years (*p* = 0.0009) and survival (Table 5). Although the overall COX-2 expression was 52.1%, the COX-2 expression by HGSC or type II carcinomas of 72% (18 of 25 cases) from the current study is in agreement with their study (*p* = 0.0038 for grade and 0.0097 for type II tumours). The expression of COX-2 and its downstream molecules may represent important targets for the development of anti-tumour therapies with possible limitations to certain histological sub-types and grades. Furthermore, some studies found no association between COX-2 over-expression and age, histological type or histological grade [7, 28, 29]. However, other previous studies have shown associations between COX-2 over-expression and age of patients, FIGO stage or histology type, and this may also explain the heterogeneous results of COX-2 expression [31–33]. Expression of COX-2 is marked in EOCs which is even stronger in patients younger than 60 years [31, 34]. However, Tiina-Liisa *et al* [30] had a contrary observation by demonstrating a statistical association between COX-2 expression and age above 57 years. In this current study, there was no association between COX-2 and age.

In addition, the result of COX-2 heterogeneity may be affected by histological sub-type. From an epidemiological point of view, reduction in mortality from post-diagnosis use of NSAIDs is strongly associated with serous ovarian cancer which is the predominant histological subtype [13]. Among a smaller number of patients with a non-serous tumour, post-diagnosis non-aspirin NSAID use was associated with increased ovarian cancer mortality [13]. Some studies found higher COX-2 expression in non-mucinous than in mucinous tumours [32]. Also, a study by semi-quantitative PCR method has indicated that serous and endometrial tumours had higher COX-2 expression, while clear cell carcinomas had lower COX-2 levels [33]. However, higher COX-2 over-expression was observed to be associated with non-serous and type I tumours [31]. COX-2 expression had also been found to be markedly elevated in well-differentiated tumours (*p* 0.0041) and had no association with histological types I and II. In the present study, COX-2 over-expressions correlate with high-grade carcinomas (*p* 0.00382), and type II histological type or HGSC (*p* 0.0097) but no such association with age. In terms of region of study, higher COX-2 expression had a poor prognosis in European and Asian studies and this may reflect the genetic constituents of patients of which the information is lacking in Africa [35]. COX-2 gene polymorphism may be key in this regard. COX-2 gene Polymorphisms such as 765 CG, 1,195 AA genotype and 765 C alleles increase EOCs risk while AG genotype and G allele of −1,195 gene decrease the risk [6].

There are possible explanations why COX-2 expression rate is relatively low in many studies despite evidence suggesting COX-2 involvement in every stage of carcinogenesis and chemo-preventive effects of NSAIDS. First, some of the EOCs lacking the COX-2 expression over-expressed COX-1 and thus, dysregulation in COX-1 and COX-2 expression is found in a higher percentage of EOCs [7, 31]. Interestingly, Both COX-1 and COX-2 were expressed in surface ovarian epithelium lining inclusion cysts (one of the early phases in tumourigenesis), thus suggesting an early sign of carcinogenesis [1]. Second, any alteration in up- or down-streams of COX-2 (arachidonic acid metabolism) pathways can mimic the cellular effect of COX-2. Third, COX-2 over-expression in stromal cells has been described [36]. The stromal expression of COX-2 may mean that a significant number of EOCs may be under the influence of COX-2 in a paracrine fashion. Furthermore, observation has shown that some ovarian cancer cells in cell culture that do not express COX-2 may probably be due to the absence of their basement membranes as well as the surrounding and supporting stromal cells that support this paracrine regulation [36]. These observations of COX-2 expression in tumour and stromal cells, and co-expression COX-2 with COX-1 may potentially be the targets for chemoprevention and chemotherapy with molecules targeting COX-2 metabolism, including NSAIDs. As a sequel to this study, a wide clinical trial of NSAIDS may be carried out if the available data in the near future justifies it. Based on sufficient data, the USA and Australia have approved the use of NSAIDs for the chemoprevention of colorectal cancer [14, 15].

### Strength and weakness

This study gives a better understanding of EOC in Nigeria and attenuates the racial or regional disparity in terms of COX-2 data availability in Africa from other regions. However, multicentre studies across Africa will give a better reflection of COX-2 expression in EOC in Africa, and this underlies the weakness of this study. This will also give a better understanding between COX-2 expression and the uncommon non-serous EOCs in Africa.

### Implications for practice and future research

High burden of EOCs in premenopausal women tasks researchers and policy makers on the need to develop actions on prevention and efficient management of EOC. The expression of COX-2 and its downstream molecules may represent important targets for the prevention and treatment of EOCs but attention needs to be paid to certain sub-types or grades. This implies that combination therapy with NSAIDs may yield a good response to chemotherapy or surgery. This study, therefore, provides a strong rationale for additional studies that may be helpful in the prognosis of ovarian carcinomas and COX-2 targeted therapy and chemoprevention.

## Conclusion

More than one-third (42.3%) of EOCs occurred ≤50 years, and the majority of cases are mostly between 40 and 69 years which corresponds to the peak of high-grade tumours. Over half (52.1%) of EOC over-expressed COX-2. There were significant statistical associations between COX-2 over-expression and grade, type II tumours or high-grade serous tumours but not with age indicating that it may influence outcomes and prevention of EOCs with possible variation in tumour type and grade.

## Conflicts of interest

The authors have declared that no competing interests exist.

## Funding

The authors received no specific funding for this work.

## Figures and Tables

**Figure 1.**
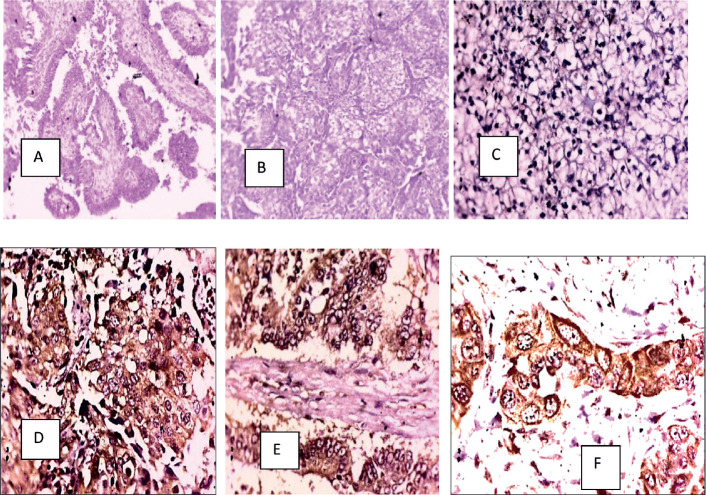
(a): HGSC (H&E), (b): Clear cell carcinoma (H&E), (c): COX-2 negative in a clear cell carcinoma, (d): COX-2 positive in a HGSC, (e): COX-2 positive in a mucinous carcinoma and (f): COX-2 positive in a serous carcinoma.

**Table 1.** Showing COX-2 IRS system and the distribution of IRS scores among 48 EOCs.

Intensity(I) expression*		Distribution (D)		IRS (I × D)		COX-2
Category Non Weak Moderate Strong -	Score 0 1 2 3 -	Category Non 1%–10% 11%–50% 51%–80% >80%	Score 0 1 2 3 4	Category Negative Weak positive Moderate positive Strong positive -	Score 0 1–4 5–8 9–12 -	Expression Negative Negative Positive Positive -
IRS	IRS (0)	IRS (1–4)	Moderate IRS(5–8)	Strong IRS(9–12)	Percentage of overall positive	Percentage of strong positive
Carcinomas Low grade serous High grade serous Mucinous Clear cell Carcinosarcoma Transitional cell	3 1 3 1 - -	4 7 3 - - 1	1 10 1 - - -	1 8 2 1 1 -	2/9 (50) 18/26 (69.2) 3/9 (33.3) 1/2 (50) 1 (100) 0	1/9 (11.1) 8/26 (30.8) 2/9 (22.2) 1/2 (50) 1 (100) 0
Total (%)	8 (16.7)	15 (31.2)	12 (25)	13 (27.1)	21/48 (43.8)	

* COX-2 positive case is defined by IRS score of 5–12. IRS is immune-reactive score

**Table 2.** Age of patients with 52 EOCs with histological sub-type and tumour grade.

Age group (years)	20–29	30–39	40–49	50–59	60–69	70–79	Total (%)	*p* value
Histology sub-type								
Low grade serous	0	2	0	2	3	2	9	0.10689
High grade serous	3	0	6	8	6	3	26	
Mucinous	1	2	3	3	0	0	9	
Clear cell	1	1	0	0	1	0	3	
Endometrioid	0	0	2	0	0	0	2	
Transitional cell	0	0	0	0	0	1	1	
Malignant Brenner	0	0	0	0	1	0	1	
Carcinosarcoma	1	0	0	0	0	0	1	
Total	6	5	11	13	11	6	52 (100)	
								
Type I EOC	3	5	3	5	5	3	24 (46.2)	0.16793
Type II EOC	3	0	8	8	6	3	28 (53.8)	
Total	6	5	11	13	11	6	52 (100)	
								
Grade High	5	2	9	6	7	3	32 (61.5)	0.33095
Low	1	3	2	7	4	6	20 (38.5)	
Total	6	5	11	13	11	9	52 (100)	

**Table 3.** Histological sub-types of 52 EOC and their grades.

Carcinomas	High grade	Low grade	Total
Low grade serous High grade serous Mucinous Clear cell Endometrioid Transitional cell Malignant Brenner tumour Carcinosarcoma	0 26 0 3 2 0 0 1	9 - 9 - 0 1 1 0	9 26 9 3 2 1 1 1
Total (%)	32(61.5)	20(38.5)	52(100)

**Table 4.** COX-2 expression with age group, tumour grade and histological sub-type of 48 EOCs.

Categories		COX-2 over-expression	COX-2 negativity	X^2^ value	df	*p* value
Age group	20–29 30–39 40–49 50–59 60–69 70–79	2 3 5 7 5 3	4 1 4 6 5 3	1.77445	5	0.87939
Grade	Low grade (19) High grade (29)	*5 20	14 9	8.36676	1	0.00382
Sub-type	Low grade serous High grade serous Mucinous Clear cell Carcinosarcoma Transitional	2 18 3 1 1 0	7 8 6 1 0 1	9.55719	5	0.0888
	Type I Type II	7 18	15 8	6.68351	1	0.00973

**Table 5.** COX-2 studies on EOCs and their main characteristics.

Study/method/country	Sample size	Mean Age	Sub-type	Positive (%)	Over-expression cut-off point
Africa					
Current study, 2024 (Nigeria)	48	50	Serous, others	25 (52.1)	IRS 5–12(≥6)
America					
Ali-Fehmi *et al* [37] 2011, IHC (USA)	126	57.6	Serous	96 (76.2)	I ≥2 and D >10%, or I ≥1 and D >50%
Khalifeh* et al* [38] 2004, IHC (USA)	96	62	Serous	65 (67.7)	I: 2 or 3 and D >10% or I: 1,2 or 3 and D >50%
Lee *et al* [39] 2006, IHC (USA)	54	51	Serous, mucinous, clear cell, endometrioid	42 (77.8)	I:2 or 3 and D>10% or I:1and D >50%
Europe					
Ferrandina *et al* [28] 2002, IHC (Italy)	87	57	Serous, others	39 (44.8)	I≥2, and D>10%
Raspollini *et al* [40] 2004, IHC(Italy)	78	58	Serous	54 (69.2)	I≥2, and D>10%
Ferrero *et al* [41] 2011, IHC, (Italy)	113	62	Serous,mucinous, endometrioid, undifferentiated	45 (39.8)	I≥2, and D>10% or I≥1, and D>50%
Surowiak *et al* [42] 2006, IHC (Poland)	43	NA	Serous, endometrioid	19 (44.2)	Stained in cell clumps or all tumours cells
Tiina-Liisa *et al* [30] 2004, IHC (Finland)	442	57	Serous	310 (70.1)	D >10%
Athanassiadou *et al* [43] 2008 , IHC (Greece)	100	62	Serous,mucinous, endometrioid,undifferentiated	56 (56)	D >10%
Denkert *et al* [34] 2002, IHC (Germany)	86	NA	Serous, others	36 (42)	Diffuse, or focal stain
Magnowska *et al* [29] 2014 (Poland)	65	NA	Serous, others	33 (50.8)	IRS >6
Asia/Europe					
Taskin *et al* [44] 2012 , IHC Turkey	32	58.63	Serous	15 (46.9)	Product of I and D >3
Ozuysal *et al* [45] 2009, IHC (Turkey)	44	54.2	Serous	17 (38.6)	I≥2 and D >10% or I≥1 and D >50%
Asia					
Seo *et al* [32] 2004, IHC (South Korea)	64	51	Serous, endometrioid, mucinous	31 (48.4)	D>5% for mucinous or D>30% for serous and endometrioid
Wang *et al* [46] 2011, IHC (China)	147	43.15	Serous, others	109 (74.1)	I≥2 or D ≥ 30%
Lou *et al* [47] 2004, IHC (China)	70	54	Serous, others	42 (60)	D >10%
Fujimoto *et al* [48] 2006, ELISA (Japan)	60	NA	Serous,mucinous,endometrioid	30 (50)	>14 ng/mg protein

I = Stained intensity on tumours cells, D = distribution of stain among tumour cells (proportion of tumour cells that stained with COX-2), IRS = immuno-reactive score, IHC = immunohistochemistry, ELISA = enzyme-linked immunosorbent assay, NA = not available
